# Closure of Non‐malignant Acquired Tracheoesophageal Fistula With Anterolateral Thigh Free Flap: A Case Report

**DOI:** 10.1002/oto2.70088

**Published:** 2025-02-20

**Authors:** Sindhura Sridhar, Aatin K. Dhanda, Nadia G. Mohyuddin, Joshua J. Kain

**Affiliations:** ^1^ Texas A&M University College of Medicine Houston Texas USA; ^2^ Department of Otolaryngology–Head and Neck Surgery Houston Methodist Hospital Houston Texas USA

**Keywords:** anterolateral thigh, free flap, microvascular reconstruction, tracheoesophageal fistula

Tracheoesophageal fistulae (TEF) are pathologic connections formed between the trachea and esophagus.^1^ In adults, TEF is a rare acquired condition presenting with dysphagia, recurrent pulmonary infections, and respiratory distress.^1^ Acquired TEF carries high risk of mortality due to complications of pulmonary infection and interference with nutrition.^1^ Surgical management is required, with common approaches including direct suture closure, tracheal resection and anastomosis, esophageal diversion, and local muscle flaps.^2^, ^3^ We present novel use of an anterolateral thigh (ALT) free flap to repair acquired nonmalignant TEF. This study was approved by the Houston Methodist Hospital Institutional Review Board (No. PRO00032976, approved 06/20/22). The patient provided written consent for the publication of study data.

## Case Report

**Figure 1:**
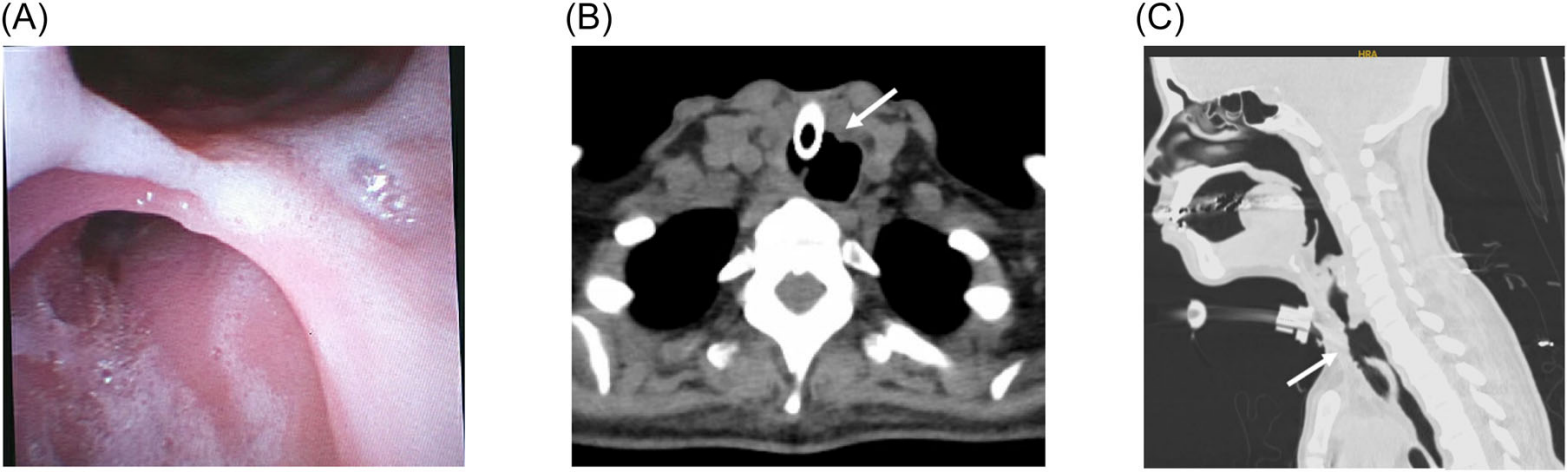
(A) Tracheoesophageal fistulae (TEF) visualized via bronchoscopy. (B, C) Axial and sagittal view of TEF on Computed tomography.

A 55‐year‐old female was admitted for atrial fibrillation with rapid ventricular rate secondary to thyrotoxicosis and acute pulmonary edema, requiring endotracheal intubation and mechanical ventilation. Tracheostomy was performed three weeks after admission for acute hypoxic respiratory failure secondary to MSSA pneumonia. Over the next month, she was unable to tolerate oral feeding due to oropharyngeal dysphagia and recurrent aspiration. Computed tomography (CT) imaging demonstrated TEF and the distal tracheostomy canula in the esophagus. The TEF was confirmed by bronchoscopy in the proximal subglottic trachea (Figure 1A). The defect measured 1.4 × 1.7 cm in aperture and 3 cm in craniocaudal length (Figure 1B,C).

**Figure 2:**
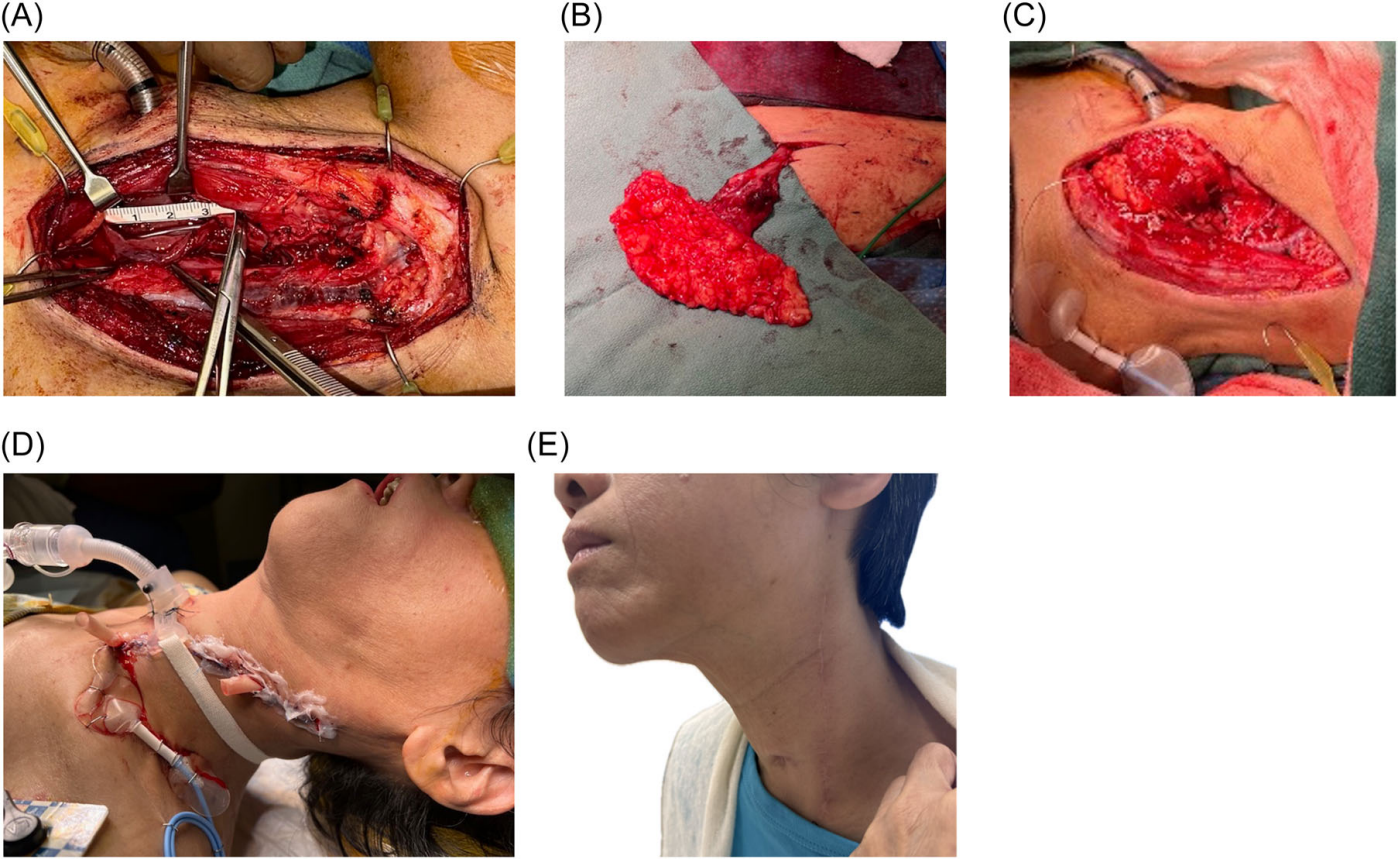
(A) Dissected tracheoesophageal fistulae (TEF), craniocaudal length 3 cm. (B) Dissected ALT free flap with descending circumflex vessels in situ. (C) Flap inset and reperfused after microvascular anastomosis. (D) Immediate postoperative view of the patient. (E) Well‐healed external excision 11 months postoperation.

The patient underwent left neck exploration and TEF repair with an ALT free flap. The left neck was dissected until the tracheoesophageal groove was visualized. Separation of the laryngotracheal complex and cervical esophagus revealed the fistula (Figure 2A). A single‐perforator fasciocutaneous ALT flap was harvested from the left leg and primary closure of the donor site was performed (Figure 2B). The fascial surface of the flap was inset circumferentially within the fistula site along the trachealis margin. The adipose surface of the flap was sewn to the esophageal myomucosal margin of the fistula mouth and the remaining paddle was sewn to the pre‐vertebral fascia. The superior thyroid artery and middle thyroid vein were anastomosed to the flap pedicle (Figure 2C). Microvascular arterial anastomosis was performed between the descending circumflex femoral artery and superior thyroid artery. A 2.0 mm coupler was used between the dominant vena comitante of the flap and middle thyroid vein. The neck was closed in layers with placement of two passive Penrose drains. After closure, the endotracheal tube was replaced with a cuffed flexible tracheostomy tube (Figure 2D).

The patient was monitored for two weeks postoperatively and had an uncomplicated hospital course. Three weeks postoperatively, her tracheostomy was decannulated and she was able to advance to full oral intake 1 month postoperatively. Around this time, she experienced intermittent tracheal stenosis due to formation of granulation tissue. She underwent four endoscopic dilations with laser excisions of stenotic tissue. The stenotic segment was cultured and treated with inhaled and systemic steroids as well as culture‐directed antibiotics. Currently, the patient undergoes monthly bronchoscopies and the mucosa at the prior site of stenosis has stabilized. The flap reconstruction healed well and there is no evidence of recurrent fistula at 11 months (Figure 2E).

## Discussion

Acquired nonmalignant TEF is a rare but dangerous complication, occurring in 0.5% of patients who undergo tracheostomy.^3^ Our patient had multiple risk factors for TEF development including thyrotoxicosis, long‐term ventilation, MSSA pneumonia, corticosteroid use, and protein‐calorie malnutrition.

Free‐tissue transfer is a well‐established reconstructive option for TEF repair with radial forearm free flaps having been described for repair of malignant, postradiated, and acquired nonmalignant TEF.^4^, ^5^ In these patients, local tissue may not be suitable for repair, or the defect may be too complex to close with limited tissue. Since our patient was cachectic with poor tissue thickness and quality at the forearm site, we used a de‐epithelialized ALT free flap trimmed in the supra‐fascial plane to improve vascularization at the repair site and maximize the chance of successful repair. Although the patient required post‐operative management of tracheal stenosis, there was no fistula recurrence and she was able to breathe comfortably and resume oral intake within a month of repair.

We describe a case of acquired nonmalignant TEF repaired with ALT free flap, a novel application of a previously described reconstructive technique. We affirm that free flaps are a viable alternative to local muscle flaps for TEF repair and reconstruction.

## Author Contributions

**Sindhura Sridhar**, manuscript writing—original draft; study conception and design. **Aatin K. Dhanda**, manuscript writing—review and editing; study conception and design. **Nadia G. Mohyuddin**, manuscript writing—review and editing. **Joshua J. Kain**, manuscript writing—review and editing; supervision; study conception and design.

## Disclosures

### Competing interests

The authors declare no conflict of interest.

## Funding source

No financial support was received.

## References

[1] Kim HS , Khemasuwan D , Diaz‐Mendoza J , Mehta AC . Management of tracheo‐oesophageal fistula in adults. Eur Respir Rev. 2020;29(158):200094. 10.1183/16000617.0094-2020 33153989 PMC9488631

[2] Marulli G , Mammana M , Natale G , Rea F . Surgical treatment of acquired benign tracheoesophageal fistulas. J Vis Surg. 2018;4:123. 10.21037/jovs.2018.06.07

[3] Mathisen DJ , Grillo HC , Wain JC , Hilgenberg AD . Management of acquired nonmalignant tracheoesophageal fistula. Ann Thorac Surg. 1991;52(4):759‐765. 10.1016/0003-4975(91)91207-C 1929626

[4] Cohen WG , Chalian A , Brody RM . Flap‐based closure of acquired tracheoesophageal fistulas. Laryngoscope. 2024;134:3761‐3764. 10.1002/lary.31386 38466164

[5] Wein RO , Popat SR , Watson T , Orlando G . Management of an acquired tracheoesophageal fistula with a fascial free flap. Head Neck. 2002;24(6):609‐613. 10.1002/hed.10076 12112560

